# Microstructure Evolution and In Situ Resistivity Response of 2196 Al-Li Alloy during Aging Process

**DOI:** 10.3390/ma16237492

**Published:** 2023-12-03

**Authors:** Xiang Li, Hongying Li, Haoqing Tang, Xiang Xiao, Jiaqiang Han, Ziqiao Zheng

**Affiliations:** 1 School of Materials Science and Engineering, Central South University, Changsha 410083, China; lixiang20231011@163.com (X.L.); 213112148@csu.edu.cn (H.T.);; 2 China Aluminum Materials Application Research Institute Co., Ltd., Beijing 102209, China; xiang_xiao@chalco.com.cn (X.X.);; 3 State Key Laboratory of Lightweight High−Strength Structural Materials, Central South University, Changsha 410083, China

**Keywords:** 2196 Al-Li alloy, in situ resistivity, microstructure, aging

## Abstract

The microstructure evolution of 2196 Al-Li alloy during aging was investigated by microhardness test, transmission electron microscope (TEM) analysis and in situ resistivity measurement. The results showed that the resistivity of the 2196 Al-Li alloy during aging rapidly decreased during the first few hours, and then gradually increased after reaching the minimum value, which is temperature−dependent. The microstructure of the alloy was dominated by the δ′ phase after aging at 160 °C for 2 h while the T_1_ phase could hardly be seen until it had been aged for 16 h. As the aging time went on, significant ripening appeared for the δ′ phase while typical growth could be observed for the T_1_ phase. The increase in the resistivity of the 2196 Al-Li alloy during aging was attributed to the stronger electron scattering capacity of the T_1_ precipitation and the coupling effect between the T_1_ and δ′ phases.

## 1. Introduction

Developments in the aerospace industry have put forward higher requirements for structural parts with lightweight and comprehensive performance. The third aluminum−lithium alloy has drawn wide attention for its low density, high specific strength and modulus, and good mechanical properties [1,2,3]. The 2196 Al-Li alloy with a high Li/Cu ratio shows a higher Young’s modulus allowing extra weight reduction in addition to lower density. The 2196−T8511 extrusions are particularly suited to stiffening the damage−tolerant fuselage and lower wing skin. It is also used in buckling applications and recommended for inner structural parts such as floor beams and seat tracks. To date, it has been widely used in aircraft components such as A380 aircraft cockpit beams and C919 long trusses, floor beams, pillars and seat rails, etc., [4,5]. Unlike the Al−Cu−Li alloys with a lower Li/Cu ratio which can be considered as having T_1_ as the dominant phase and not forming any δ′ phase, the 2196 alloy has a mixed T_1_/δ′ microstructure in the peak−aged state [6].

Electrical resistivity is a microstructure−sensitive parameter because electron scattering can be significantly influenced by the decomposition of supersaturation solid solutions and the quantity, volume fraction, morphology and distribution of precipitates [7,8,9,10,11,12]. Therefore, the microstructure evolution of an alloy during aging can be reflected by the electrical−resistivity variation. This has been fully confirmed by the electrical conductivity studies on 6xxx Al−Mg−Si alloys [13,14,15,16,17,18]. For instance, Esmaeili et al. [15] studied the resistivity change in AA6111 aluminum alloy during artificial aging. The results showed that the higher the aging temperature, the faster the resistivity of the alloy decreased. Khangholi et al. [17] investigated the effect of Mg/Si ratio and aging treatment on the electrical conductivity of an Al−Mg−Si 6201 alloy. It was found that Si solutes are the main factor for the conductivity. Another work by Khangholi et al. [18] quantitatively studied the effect of β’ phase on the resistivity of the AA6201 alloy. It was shown that the larger number density and the smaller size of the β’ phase contribute more significantly.

For the 2196 Al-Li alloy, comparable amounts of spherical δ′ phase and plate−like T_1_ phase exist in the peak−aged state [19,20,21], giving the alloy a complex aging precipitation with two precipitation sequences as a function of the aging time. In addition, the coupling effect on the scattering of electrons by the coexistence of aging precipitates with different morphologies has rarely been considered. In this paper, to further clarify the microstructure evolution of the 2196 alloy during aging, a novel in situ resistivity measurement which can monitor the resistivity response continuously during aging was employed. This self−designed setup has been verified in other works [22,23]. The microstructure evolution was characterized by transmission electron microscopy (TEM). Finally, the mechanism of coexistence of the δ′ and T_1_ phases on the resistivity response was further discussed. Last but not least, since the corrosion resistance of an alloy is closely related to the electrical conductivity, the continuous resistivity measurement in this work can also be used as a reference for corrosion−resistance evaluation. It should be noted that the result in the present work was consistent with the corrosion characteristic of the Al−Cu−Li alloy that displays maximum resistance to IGC/IGSCC in peak−aged condition, with enhanced susceptibility in under−aged and over−aged conditions [24].

## 2. Experiment and Methods

The alloy used in this study is the extrusion profile of 2196 Al-Li alloy, which has the nominal composition indicated in Table 1. Initially, the samples were solution treated at 540 °C for 60 min and water quenched. The aging treatment was conducted at 160 °C, 180 °C and 200 °C, respectively.

A 310HVS−5 Vickers microhardness tester (Laizhou Huayin Test Instrument Co., Ltd., Laizhou, Shandong, China) was used to measure the hardness and each sample was measured five times to obtain the average value. The resistivity of the alloy after aging was measured off−line using a Sigma2008 eddy current digital conductivity tester (Xiamen Tianyan instrument Co., Ltd., Xiamen, Fujian, China). Each sample was measured 10 times and the average value was taken.

**Figure 1:**
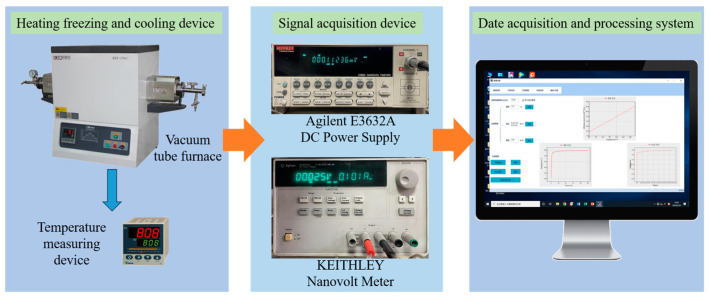
Schematic diagram of the in situ resistivity measuring system.

To monitor the continuous change in the resistivity of the alloy during aging, the deliberately designed in situ measurement setup was employed, the schematic of which is shown in Figure 1. From Figure 1, it can be seen that the in situ measurement setup consisted of three modules. In the heating, freezing and cooling module, one tube furnace was used to control the heating and dwelling process. In the signal acquisition module, the Agilent E3632A direct current power supply (Beijing Jinlong Yiyang Science&Technology Co., Ltd., Beijing, China.) was used to provide current for the loop and a test current of 100 mA, and the KEITHLEY nanovoltmeter (Tektronix (China) Inc., Shanghai, China) was used to collect the voltage signal every 3 s. In the data acquisition and processing module, converting the signal and exporting the in situ resistivity values was carried out through the software. Each aging condition was tested at least three times to obtain the average value, and the uncertainty was 2%.

**Figure 2:**
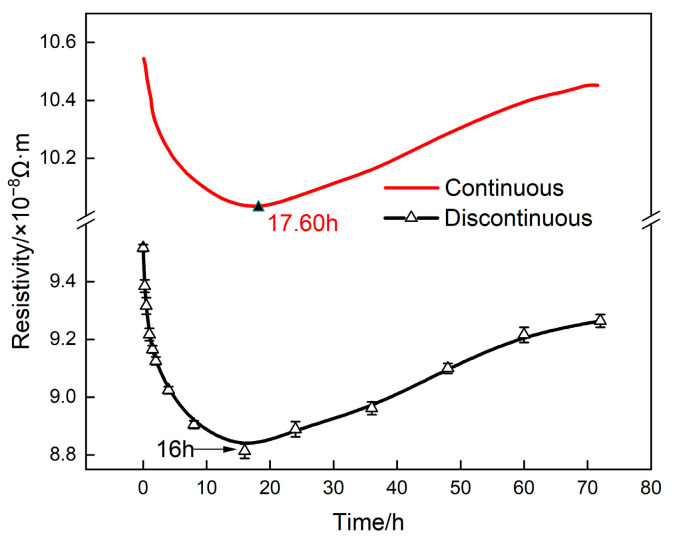
Resistivity of 2196 aluminum alloy aged at 160 °C.

To verify the reliability of the in situ measurement device, a comparative analysis was conducted on the continuous in situ and off−line resistivity measurement of the alloy aged under 160 °C, as shown in Figure 2. From Figure 2, it can be seen that the continuous in situ measurement results were highly consistent with those of the off−line measurements. It is worth noting that the higher measured value of the continuous measurements was attributed to the fact that the in situ measurement was conducted at elevated temperature.

Samples for TEM observations were prepared by electropolishing in a twin−jet apparatus using a 20% nitric acid and methanol solution. Conventional TEM observation was conducted on a Tecnai G2 20 microscope (FEI Company, Hillsboro, OR, USA) and a convergent−beam electron diffraction (CBED) method was used to measure the thickness of the TEM samples. The size, number density and volume fraction of precipitates were statistically analyzed using Image J 1.53 software and 15 fields of view were selected for each sample.

## 3. Results and Discussion

### 3.1. Resistivity Response during Aging

**Figure 3:**
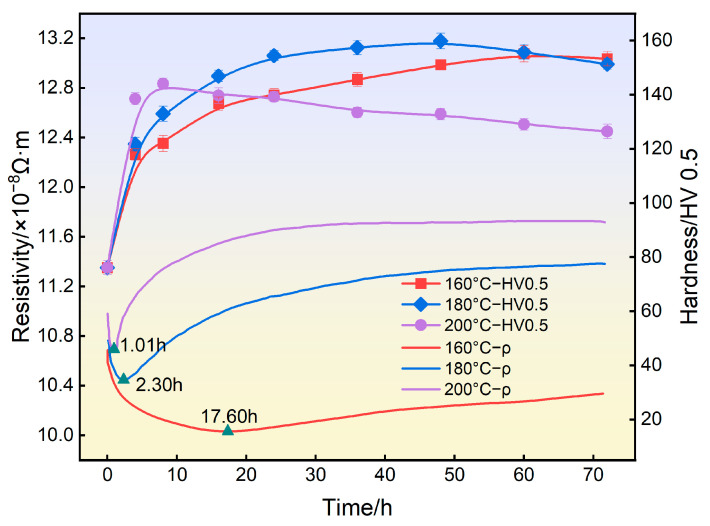
Hardness and in situ resistivity curves of 2196 alloy aged at different temperatures.

Figure 3 shows the hardness evolution and in situ resistivity response of 2196 Al-Li alloy during aging at different temperatures. From the hardness curves, it can be seen that in first few hours, a very steep increase happened. When the aging temperature was 160 °C, the hardness curve reached the maximum value at 155.1 HV after 60 h. When the aging temperature was 180 °C, the hardness curve of the alloy reached 158.2 HV after 48 h and then decreased slightly. When the aging temperature was 200 °C, the hardness of the alloy rapidly reached the peak value of 144.0 HV after 8 h and then significant over−aging could be observed.

From the in situ resistivity response curves, it can be seen that when aging at 160 °C, the resistivity of the alloy first decreased quickly, and then increased slowly after reaching the minimum value at 17.60 h. When aging at 180 °C, the resistivity of the alloy reached the minimum value at 2.30 h and then increased rapidly. When aging at 200 °C, the resistivity of the alloy reached the minimum value only at 1.01 h and then rose rapidly, and finally tended to be stable.

### 3.2. Microstructure Evolution

**Figure 4:**
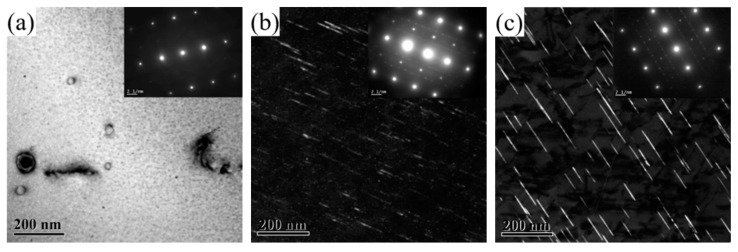

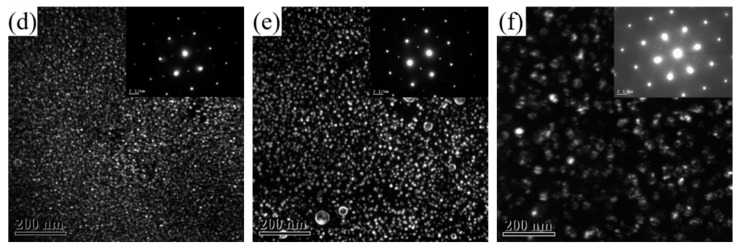
TEM images of 2196 alloy aged at different temperatures for 2 h: (**a**) 160 °C <112>_Al_; (**b**) 180 °C <112>_Al_; (**c**) 200 °C <112>_Al_; (**d**) 160 °C <110>_Al_; (**e**) 180 °C <110>_Al_; (**f**) 200 °C <110>_Al_.

Figure 4 shows the TEM images of the 2196 Al-Li alloy aged for 2 h at three different temperatures. The zone axis for Figure 4a–c is <112>_Al_ while that for Figure 4d–f is <110>_Al_. From Figure 4a,d, it can be seen that the δ′ phase dominated the microstructure when the alloy had been aged for 2 h at 160 °C. It should be noted that both the bright−field TEM image and selected area electron diffraction (SAED) in Figure 4a,d showed no signal of T_1_ precipitation. When aged for 2 h at 180 °C, as can be seen from Figure 4b,e, the number density decreased and the size of the δ′ phase increased, accompanied by the appearance of the T_1_ phase, which can be observed from Figure 4b along the <112>_Al_ zone axis. When aged for 2 h at 200 °C, a large amount of T_1_ phase could be found while the δ′ phase showed significant ripening with decreased number density.

After aging for 2 h at 160 °C, no T_1_ phase appeared in the alloy, and the resistivity did not reach the minimum value at this time. After aging for 2 h at 180 °C, a small amount of T_1_ phase could be observed in the alloy, and the resistivity dropped to near the minimum value at this time. After aging for 2 h at 200 °C, certain amounts of T_1_ phase appeared in the alloy, and the resistivity had already reached the minimum value and started to rise. In general, the precipitation of the T_1_ phase halted the decrease in the resistivity of 2196 alloy as the aging further progressed.

**Figure 5:**
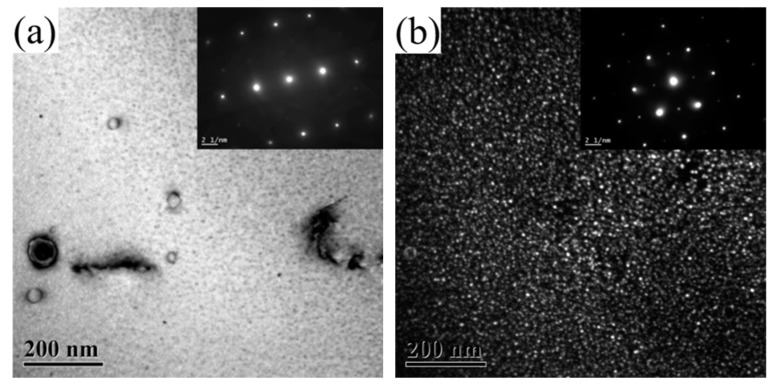

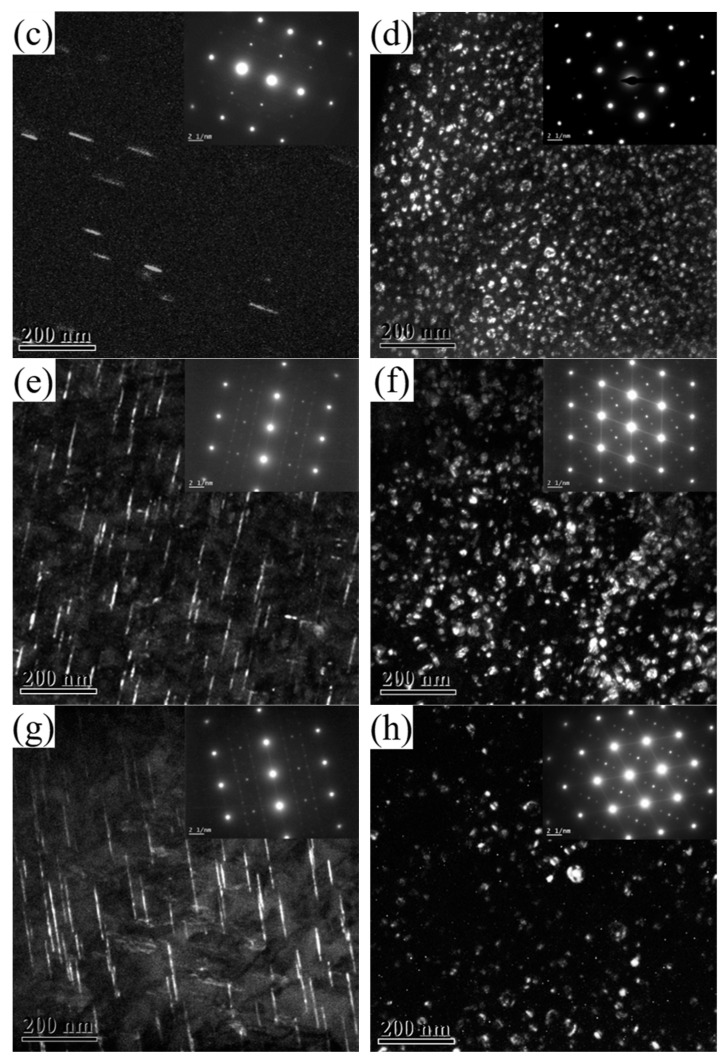
TEM images of 2196 alloy aging at 160 °C for different times: (**a**) 2 h <112>_Al_; (**b**) 2 h <110>_Al_; (**c**) 16 h <112>_Al_; (**d**) 16 h <110>_Al_; (**e**) 48 h <112>_Al_; (**f**) 48 h <110>_Al_; (**g**) 60 h <112>; (**h**) 60 h <110>.

Figure 5 shows the TEM images of 2196 alloy aged at 160 °C for 2–60 h. The zone axis for Figure 5a,c,e,g was <112>_Al_ while that for Figure 5b,d,f,h was <110>_Al_. As mentioned above, when the alloy aged at 160 °C for 2 h, a large amount of δ′ phase dispersed in the alloy but the T_1_ phase did not appear. This means that the aging precipitation at this time is only composed of δ′ phase. When the alloy had aged for 16 h, as can be seen from Figure 5c,d, the distribution of T_1_ phase could occasionally be found in the matrix while the diameter of the δ′ phase significantly increased. This means that the aging precipitation in the alloy at this time was composed of a few T_1_ phase and a large number of grown−up δ′ phase. At the same time, it should be noted that the resistivity of the alloy dropped to near the minimum value. When the alloy aged for 48 h and 60 h, as can be seen from Figure 5e–h, in addition to a larger number of T_1_ phase being found in the matrix, obvious coarsening with reduced number density and increased diameter had taken place for the δ′ phase.

When the δ′ phase precipitated, the solute contents decreased due to the decomposition of Li atoms, and the scattering effect of solute atoms on electrons weakened. Therefore, in the initial stages of aging, the resistivity of the alloy rapidly decreased. When a small amount of T_1_ phase appears in the alloy, the resistivity tends to increase with the further precipitation of the T_1_ phase. This can be explained by the T_1_ phase having a greater ability to scatter electrons than the δ′ phase. With the progress of aging, the solute content in the matrix decreases, and hence the driving force for aging precipitation decreases. Therefore, the slope of the resistivity curve gradually decreased in the later stage of aging.

### 3.3. Relationship between Resistivity Response and Aging Precipitation

In order to better illustrate the relationship between the aging precipitation and the resistivity response of the 2196 alloy, the Matthiessen law is introduced; that is, the total resistivity of an alloy is the sum of contributions from many microstructure features to electron scattering [18], as expressed by Equation (1):

(1) $$\rho_{\mathrm{total}}=\rho^{\mathrm{PURE}}+\rho^{\mathrm{DISLO}}+\rho^{\mathrm{GB}}+\rho^{\mathrm{PREC}}+\rho^{\mathrm{SOL}}$$

where $\rho^{\mathrm{PURE}}$ is the intrinsic resistivity of pure aluminum at room temperature, 2.79 × 10^−8^ Ω·m. $\rho^{\mathrm{DISLO}}$, $\rho^{\mathrm{GB}}$, $\rho^{\mathrm{PREC}}$ and $\rho^{\mathrm{SOL}}$ are the resistivity from dislocation, grain boundary, precipitate and solute atom, respectively.

Raeisinia et al. [25] proposed a modified Matthiessen law considering the electron scattering from aging precipitation, as shown by Equation (2):

(2) $$\rho_{\mathrm{total}}=\rho^{\mathrm{pure}}+L^{\mathrm{dislo}}{\Delta \rho }^{\mathrm{dislo}}+S^{\mathrm{gb}}{\Delta \rho }^{\mathrm{gb}}+\frac{{\Delta \rho }^{\mathrm{prec}}}{{\left(L^{\mathrm{prec}}\right)}^{1/2}}+\sum C_{i}^{\mathrm{sol}}{\Delta \rho }_{i}^{\mathrm{sol}}$$

where $\rho^{\mathrm{pure}}$ and $\rho^{\mathrm{PURE}}$ are equivalence; ${\Delta \rho }^{\mathrm{dislo}}$, ${\Delta \rho }^{\mathrm{gb}}$, ${\Delta \rho }^{\mathrm{prec}}$ and ${\Delta \rho }_{i}^{\mathrm{sol}}$ represent the resistivity constant of dislocation, grain boundary, precipitate and solutes, respectively; $L^{\mathrm{dislo}}$ is dislocation density, $S^{\mathrm{gb}}=6/d_{\mathrm{gb}}$, $d_{\mathrm{gb}}$ is average grain size; $L^{\mathrm{prec}}={\left(\frac{2\pi \times 100}{f_{A}}\right)}^{1/2}\cdot \frac{d}{2}$ is the spacing of precipitate, $f_{A}$ is volume fraction, d is diameter of precipitate; $C_{i}^{\mathrm{sol}}$ is solute concentration in the matrix.

According to the literature [17,18,25,26], the values of ${\Delta \rho }^{\mathrm{dislo}}$, ${\Delta \rho }^{\mathrm{gb}}$ and ${\Delta \rho }^{\mathrm{prec}}$ are 2.7 × 10^−25^ Ω·m^3^, 2.6 × 10^−16^ Ω·m^2^ and 12 Ω·(nm)^3/2^, respectively. Table 2 shows the ${\Delta \rho }_{i}^{\mathrm{sol}}$ of Cu and Li solute atoms. During aging, the contribution from grain boundary and dislocation to the resistivity can be ignored [18]. According to the expression of $L^{\mathrm{prec}}$, an increase in the volume fraction and a decrease in the diameter of precipitates will lead to a decrease in the spacing between precipitates, thereby enhance the electron scattering. According to the literature [25], when the spacing of precipitates is in the order of 10 nm, the contribution to the resistivity from the precipitates reaches 15–25%. When the spacing is in the range of 10–100 nm, it accounts for 10–15%. Only when the spacing reaches the order of 1 μm, the contribution of the precipitates drops to less than 5%. In summary, the resistivity of alloys during aging depends on solutes and precipitates.

The alloy was solution treated at 540 °C for 60 min and then aged at 160 °C. The aged precipitations are mainly composed of δ′ and T_1_ phase. Table 3 shows the statistical analysis of the δ′ and T_1_ phase in 2196 Al-Li alloy aged for 2–60 h at 160 °C, where λ_δ′_ and λ_T1_ represent the diameter of δ′ phase and the length of T_1_ phase, respectively.

The number density ($N_{v}$) of δ′ and T_1_ can be calculated by Equation (3) [28].

(3) $$N_{v}=\frac{N}{A\left(t+\lambda \right)}$$

where N is the number of precipitates; A is the area of the matrix containing the precipitates; t is the thickness of TEM foil which can be obtained by CBED method and has 3% uncertainty [29]; λ is the average size of precipitates.

The volume fraction of δ′ phase and T_1_ phase can be calculated by Equations (4) and (5) [30,31,32,33]:

(4) $$f_{\delta^{\prime }}=N_{v}\cdot \frac{4\pi {\left(\frac{\lambda_{\delta ’}}{2}\right)}^{3}}{3}$$

(5) $$f_{T1}=2\cdot \frac{\frac{\pi {\left(\lambda_{T1}\right)}^{2}}{4}\cdot T_{\mathrm{avg}}}{V_{\mathrm{sample}}}$$

where $f_{\delta^{\prime }}$ and $f_{T1}$ are the volume fraction of the δ′ and T_1_ phases, T_avg_ is the average thickness of T_1_ phase, $V_{\mathrm{sample}}$ is the volume of TEM foil being observed.

**Figure 6:**
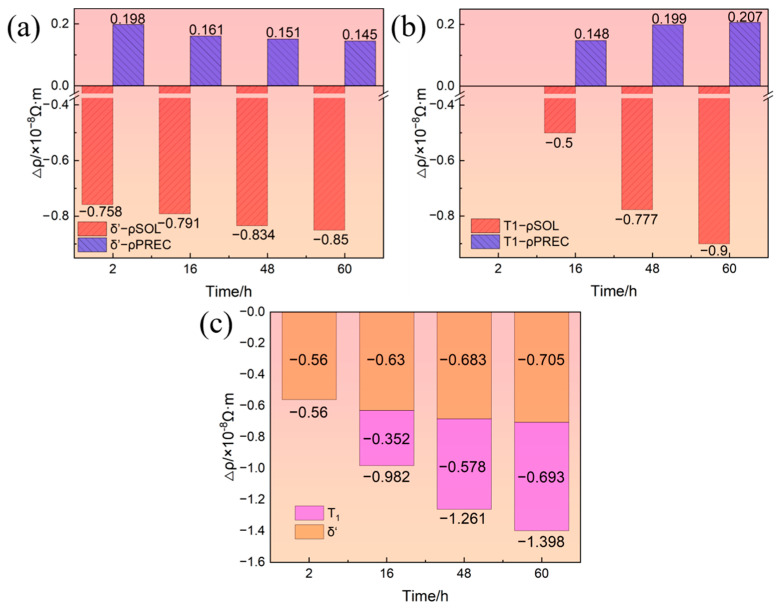
Resistivity change due to δ′ phase and T_1_ phase: (**a**) δ′ phase; (**b**) T_1_ phase; (**c**) δ′ phase and T_1_ phase.

During aging, the precipitation of the δ′ phase leads to the decomposition of Li solute while that of the T_1_ phase leads to the simultaneous decomposition of Li and Cu solutes. Therefore, the precipitation of the δ′ phase and T_1_ phase have two effects on resistivity. On the one hand, the decomposition of solute atoms leads to the decrease in resistivity. On the other hand, the precipitates of the δ′ phase and T_1_ phase lead to the increase in resistivity. Relative to solid solution state, the increase in the resistivity of the alloy due to the aging precipitates and the decrease in the resistivity of the alloy due to the decomposition of solute atoms were calculated based on the volume fraction of the δ′ and T_1_ phases, as shown in Figure 6. Figure 6a,b show the resistivity change due to the precipitation of the δ′ and T_1_ phases, respectively, where ρ^PREC^ and ρ^SOL^ represent the effect of aging precipitation and solutes on the resistivity, respectively. It can be seen from Figure 6a that as the aging progressed, the magnitude of the increase in resistivity due to the precipitation of the δ′ phase gradually decreased while that of the decrease in resistivity caused by the decomposition of Li solutes gradually increased. This is mainly attributed to the coarsening of the δ′ phase and the precipitation of the T_1_ phase causing some δ′ phases to redissolve. It can be seen from Figure 6b that the T_1_ phase had not yet precipitated after aging for 2 h. As the aging progressed, the contribution of T_1_ precipitation to the resistivity increased and the Cu and Li solutes decomposition to the resistivity decrease both gradually increased. In summary, the decrease in the resistivity of the alloy due to the decomposition of solute atoms is more significant than the increase in the resistivity of the alloy due to the aging precipitates. Therefore, the precipitation of the δ′ phase and T_1_ phase will generally reduce the resistivity of the alloy, as shown in Figure 6c. As the aging time increases, the level of decrease in resistivity caused by the precipitation of the δ′ phase increases slightly, while that caused by the precipitation of the T_1_ phase increases significantly. Therefore, the level of decrease in the resistivity caused by the precipitation of the δ′ phase and T_1_ phase becomes larger and larger.

**Figure 7:**
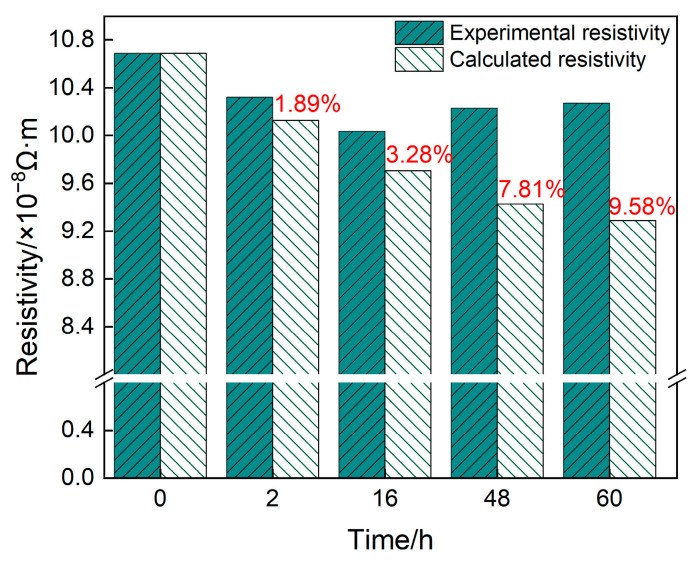
Measured and calculated values of resistivity of alloys aged at 160 °C for different times.

Figure 7 shows the absolute resistivity of the 2196 Al-Li alloy obtained by in situ measurement and theoretical calculation during aging at 160 °C for 0−60 h. From the calculated results, it can be seen that the effect of aging precipitation on the resistivity is less than that of the solutes; therefore, the resistivity of the alloy during aging gradually decreases. This is inconsistent with the in situ measurement results of this work. This gap can be attributed to the coexistence of spherical δ′ phase and plate−like T_1_ phase, which will be discussed next.

The decomposition of a supersaturated solid solution during aging will reduce the resistivity of the Al alloy. This is generally because the effect of precipitation on electron scattering is smaller than that of solid solution atoms [25]. However, the resistivity change during the decomposition of an alloy also depends on the size, spacing and shape of the precipitates. For instance, in the study of Jiang et al. [33], it was observed that the alloy with a high volume fraction of β″ phase showed lower electrical conductivity. It was considered to be the increase in electron scattering caused by the smaller precipitate spacing (~20 nm). Chen et al. [34] investigated the effect of re−solution and aging on the resistivity of an Al−Cu−Li alloy; the resistivity also appeared to increase during the aging process, which was attributed to the effect of GP zones and nanoprecipitate; in other words, it could be considered that at this time the effect of aging precipitation is greater than that of solutes.

**Figure 8:**
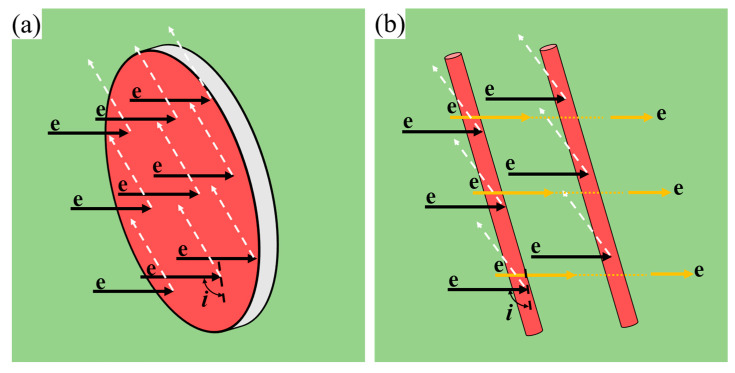
Scattering of free electrons on phases with different shapes: (**a**) lath−like; (**b**) needle− or rod−shaped.

For the 2196 alloy, the evolution of spherical δ′ phase and plate−like T_1_ phase takes place simultaneously during aging, so the corresponding resistivity response is more complicated. On the one hand, compared with the rod−like or needle−like β″ phase in Al−Mg−Si alloy, under the same electron incident angle i and incident cross−section area, the scattering effect of the plate−shaped T_1_ phase on electrons is more significant, as shown in Figure 8a,b, the arrow represents the direction of electron motion. However, the Matthiessen law has not considered the shape factor in precipitates. On the other hand, the coupling effect when multiple precipitates coexist was not taken into account in the calculation process [8]. That is, when precipitates coexist, the precipitate spacing between them is much smaller than when a precipitate phase exists alone, thus causing the resistivity to increase.

**Figure 9:**
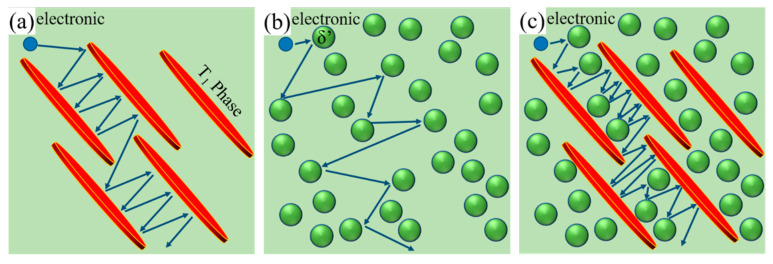
Schematic diagram of the obstruction of electron flow by single precipitating phase and the combination of precipitating phases: (**a**) T_1_ phase; (**b**) δ′ phase; (**c**) T_1_ phase and δ′ phase.

To further describe the coupling effect of the T_1_ and δ′ phase, the schematic diagram of the effects of single and coexisting δ′ and T_1_ phase on the electron scattering is shown in Figure 9, the arrow represents the direction of electron motion. Figure 9a,b illustrate the effect of single δ′ and T_1_ on electron scattering, respectively. Comparing Figure 9a with Figure 9b, it was found that the electron scattering from plate−like T_1_ phase was more significant than that of the spherical−like δ′ phase, which is consistent with the work of Gan et al. [35], who studied the effect of plate−like eutectic Si and fibrous−like eutectic Si on electron scattering. The effect of δ′ and T_1_ coexisting in electron scattering is shown in Figure 9c, from which it can be seen that the synthetic effect of δ′ and T_1_ coexisting is far beyond the simple superposition of the single δ′ and T_1_ contributions.

Combining Figure 5 and Figure 7, it can be concluded that only the δ′ phase could be observed when the 2196 Al-Li alloy was aged at 160 °C for 2 h. At this time, the deviation between the calculated and measured resistivity is only 1.89%. When the alloy was aged for 16 h, a small amount of T_1_ phase appeared and coexisted with the δ′ phase. The synthetic effect of δ′ and T_1_ made the deviation between the calculated and measured values increase to 3.28%. As the aging further progressed, the number density and size of T_1_ phase further increased. Therefore, the synthetic effect between T_1_ and δ′ phase also increased, causing a further increase in the deviation between the calculated and measured values.

## 4. Conclusions

(1) During the aging process, the electrical resistivity of the 2196 alloy decreased rapidly during the first few hours, then increased gradually. The time to minimum value was temperature−dependent; that is, the higher the aging temperature, the shorter the time to reach the minimum value (17.6 h for 160 °C, 2.3 h for 180 °C, 1.01 h for 200 °C).
(2) In the declining stage of resistivity during the aging process, the δ′ phase precipitates were the main ones in the matrix. However, in the rising stage, the T_1_ phase began to appear and was accompanied by the coarsening of the δ′ phase.
(3) The resistivity change during the aging process of the 2196 Al-Li alloy depends on the coupling effects of the T_1_ and δ′ precipitations. Different from the δ′ phase, the aging precipitation of the plate−like T_1_ phase contributes negatively to the conductivity. When T_1_ and δ′ phases coexist, there is a coupling effect on the electron scattering. As the number density and size of the T_1_ phase increase, the coupling effect becomes more significant within 60 h.

## Figures and Tables

**Table 1:** Nominal composition of 2196 alloy (wt.%).

Cu	Li	Ag	Mg	Zr	Ti	Zn	Mn
2.5–3.3	1.4–2.1	0.25–0.6	0.25–0.8	0.04–0.18	0.03	0.04	0.35

**Table 2:** Effect of solute atoms Cu and Li on the resistivity of pure aluminum [27].

Element	Maximum Solubility in Al (wt.%)	Resistivity Increment of Al per wt.%(μΩ·cm)	
		In Solution	Out of Solution
Cu	5.65	0.344	0.030
Li	4.0	3.31	0.68

**Table 3:** Statistical analysis of δ′ phase and T_1_ phase aged for different times.

Condition	δ′			T_1_		
	λ_δ′_ [nm]	N_v_ [m^−3^]	f_δ′_ [%]	λ_T1_ [nm]	N_v_ [m^−3^]	f _T1_ [%]
160 °C/2 h	6.12	3.66 × 10^23^	4.39	0	0	0
160 °C/16 h	9.5	1.02 × 10^23^	4.59	50.36	2.84 × 10^21^	1.17
160 °C/48 h	11.08	0.68 × 10^23^	4.84	63.21	1.48 × 10^21^	1.82
160 °C/60 h	12.14	0.53 × 10^23^	4.92	70.36	1.39 × 10^21^	2.11

## Data Availability

The data required to reproduce these finds cannot be shared online at this time for the ongoing study and if anyone needs it, please contact the corresponding author.
